# Investigating longitudinal context-specific physical activity patterns in transition from primary to secondary school using accelerometers, GPS, and GIS

**DOI:** 10.1186/s12966-020-00962-3

**Published:** 2020-05-18

**Authors:** Teun Remmers, Dave Van Kann, Stef Kremers, Dick Ettema, Sanne I. de Vries, Steven Vos, Carel Thijs

**Affiliations:** 1 School of Sport Studies, Fontys University of Applied Sciences, P.O. Box 347, Eindhoven, AH 5600 The Netherlands; 2 Department of Epidemiology, Maastricht, Maastricht University (Medical Center+), CAPHRI Care and Public Health Research Institute, Maastricht, the Netherlands; 3 Department of Health Promotion, Maastricht University (Medical Center+), NUTRIM School of Nutrition and Translational Research in Metabolism, Maastricht, the Netherlands; 4 Department of Human Geography and Planning, Utrecht University, Utrecht, the Netherlands; 5 The Hague University of Applied Sciences, Research group Healthy Lifestyle in a Supporting Environment, The Hague, the Netherlands; 6 Department of Industrial Design, Eindhoven University of Technology, Eindhoven, The Netherlands

**Keywords:** Context, Child, Environment, Domain, Weekday, Weekend, After school, Transport, Time segment, Walking, Cycling, Sports, Geographic

## Abstract

**Introduction:**

Previous longitudinal studies indicate that physical activity (PA) significantly declines from primary-to secondary school, and report both changes in individual and environmental determinants of PA. In order to understand this transition and to prevent this negative trend, it is important to gather contextually rich data on possible mechanisms that drive this decline. Therefore, the aim of this study was to investigate changes of PA patterns in transition between primary and secondary school, and to add domain-specific insights of how, where, and when these changes occur.

**Methods:**

In total, 175 children participated in a 7-day accelerometer- and Global Positioning System (GPS) protocol at their last year of primary and their first year of secondary school. GPS data-points were overlaid with Geographical Information Systems (GIS) data using ArcGIS 10.1 software. Based on the GPS locations of individual data-points, we identified child’s PA at home, school, local sports grounds, shopping centers, and other locations. Also, trips in active and passive transport were identified according to previously validated GPS speed-algorithms. Longitudinal multi-level linear mixed models were fitted adjusting for age, gender, meteorological circumstances, and the nested structure of days within children and children within schools. Outcome measures were minutes spent in light PA and moderate-to-vigorous PA, specified for the time-segments before school, during school, after school and weekend days.

**Results:**

Total PA significantly declined from primary to secondary school. Although transport-related PA increased before- and during school, decreases were found for especially afterschool time spent at sports grounds and transport-related PA during weekends.

**Conclusions:**

This is the first study that demonstrated longitudinal changes of context- and domain-specific PA patterns in transition between primary and secondary school, based on device-assessed PA. Given the importance of this transition-period for the development of long-term PA patterns, results from this study warrant the development of evidence-based PA programs in this transition period, while acknowledging the integrative role of schools, parents, and afterschool sports providers. More specifically, the results underline the need to increase children’s PA levels in primary schools, promote afterschool PA at secondary schools, and to prevent the drop-out in sports participation at secondary schools.

## Background

Insufficient physical activity (PA) and excessive sedentary behaviour in children have been consistently linked to various detrimental short- and long term consequences for health and general wellbeing, such as overweight and obesity [1], bone health [2], and mental health [3]. Despite this knowledge, children’s PA levels have been found to decline from childhood to adolescence [4–8].

The transition between primary and secondary school is an important phase for PA development as changes are likely to occur in children exposure to and perceptions of the physical environment (e.g. changes in PA affordances at school ground during recess), the social environment (e.g. classmate changes), and the learning environment (e.g. more academic load). To our knowledge, four studies have longitudinally investigated PA in this transition using questionnaires [9–12], or (partly) device-assessed PA [13–17]. Four additional longitudinal studies examined PA development in 9–15-year-old children, but have not explicitly investigated the change between schools [18–21]. Results from studies investigating the transition from primary to secondary school are mixed. For example, six studies reported a decline [7, 11, 12, 17, 18, 20], while two studies exhibited an increase in PA when going to secondary school [13, 16].

When studying PA during the transition between primary and secondary school, it is not only essential to accurately quantify changes in children’s total PA, but also to provide insight in possible mechanisms behind this potential change by using contextually rich data. It is also important to distinguish between several types of PA behaviour (i.e., behavioural domains), in order to understand changes in PA patterns and to provide meaningful starting points for intervention development [22]. This is because each PA domain has their own specific determinants [23, 24]. When investigating specific relationships within these behavioural domains, precise measurements of PA and the context in which PA takes place are needed [25]. Most studies measured this by complementing their device-assessed PA with self-reported data (e.g., transport mode to school) [13–15, 17]. Three studies showed an increase in self-reported duration of active transport towards secondary school [10, 15, 16], Also, several studies have suggested that changes in school environments during the transition to secondary school, are mostly explained by changes in children’s active transport [10, 13, 17, 26]. Marks et al. (2015) also showed that a change of school environment in the transition to secondary school was associated with less self-reported activity and active transport, but no differences were found on accelerometer measured daily PA [17]. This illustrates that an increase in one behavioural domain (e.g., transport-related PA) may not result in an increase in children’s total daily PA, as this may be compensated by a decrease in another behavioural domain (e.g., organized sports participation). Above described studies showed that especially transport-related PA should be considered in interpreting PA-changes in the transition between primary and secondary school. Furthermore, Marks et al. (2015) showed disagreement of self-reported active transport versus device-assessed daily PA, which is in line with numerous previous studies that showed a lack of cohesion of results from more subjective versus device-assessed PA [27, 28]. Self-reports may be especially vulnerable to social desirability or recall bias, and extensive PA dairies cause increased participant’s burden [29, 30]. To date, studies that differentiate between various PA domains using precise and more objective measures of PA are scarce [31].

The transition between primary and secondary school can be viewed as a complex interplay between changes in individual, social and environmental factors [15, 17]. Following a social-ecological perspective, PA patterns are also subject to political or cultural factors (e.g., transport-regulations of schools, or the existence of (after) school PA programs [32, 33]). In addition, the influence of potential determinants of PA depend on the context in which the activity occurs [24, 34, 35]. In order to understand mechanisms behind PA-changes in the transition phase between primary-and secondary school, it is also important to recognize the geographical context in which PA takes place and to acknowledge constraints based on time geography [24, 36]. This may give valuable insights in the comparability of studies across various cultures and contexts, and therefore foster more meaningful international comparisons. Studies that integrate geographical and temporal information to study children’s PA patterns are scarce [22, 31, 37].

Altogether, in order to understand mechanisms behind changes in PA during the transition between primary and secondary school, it is vital to accurately measure changes in PA and acknowledge a variety of contextually relevant behavioural PA domains based on information from time geography and geographical location. Studies combining accelerometers and GPS loggers may be a valuable contribution to unravel changes in specific PA domains in transition from primary to secondary school. Recent studies showed that it is feasible to passively monitor continuous PA and location data, by combining accelerometers and Global Positioning System (GPS) data [31]. When integrated with Geographical Information Systems (GIS), the behavioural domain, as well as the context of PA can be inferred from its geographical location or travel speed [38–41].

Consequently, the aim of this study was to investigate changes in PA patterns within device-assessed behavioural contexts in the transition between primary and secondary school, to add in-depth insight on how, where, and when changes in PA patterns occur. In addition, literature suggests that changes between primary and secondary school are partly explained by active transport to school. Our secondary aim was therefore to gain further insight in the role of transport-related PA during this transition.

## Methods

### Design and participants

The present study was embedded in the Physical Activity in Public Space Environments (PHASE) study, which was specifically set up to examine longitudinal relationships between characteristics of the physical environment and children’s PA patterns in the transition phase from primary to secondary schools. Children’s PA patterns were investigated at baseline (last year primary school) and follow-up (first year of secondary school). Measurements were conducted in the municipality of ‘s-Hertogenbosch, the Netherlands, which covers around 110 km^2^ flatland, and has approximately 150.000 residents [42] (see Additional file 1). Population density varies between neighbourhoods (1.8; 59.0 residents per hectare). Average population density of included neighbourhoods was 19.4 residents per hectare [43]. At baseline, we invited 30 primary schools to participate, of which 20 schools agreed. All children in their final year (approximately 1000 children) were invited to participate in our study, 341 children provided informed consent to participate in baseline measurements. One year later, all children changed schools. Participating children were approached again in secondary school for a follow-up measurement using the same protocol. Ethical approval for the PHASE study was obtained from the research ethics committee of the Maastricht University Medical Centre (reference number 12–4-077). From all participating children, the child and parents provided informed consent.

Data were collected from April till July 2015 for baseline measurements and April till July 2016 for the follow-up measurement, with an average daily temperature of 15.1 degrees Celsius (SD = 5.0) and 77% of the days with < 1.0 mm of precipitation (based on registries from a local weather station). Sunset times during this time-period were between 20:13 and 22:06 h local time in the center of the Netherlands (data extracted from http://www.timeanddate.com/sun/netherlands). Accelerometers and GPS loggers were distributed during classroom visits, where children received verbal and written instruction about how to wear the devices. Both devices were attached to the waist and worn at the right hip with a single elastic belt. Measurement protocol included wearing the devices during waking hours for 7 consecutive days, only to remove the belt during water-related activities (e.g., swimming, showering), and to recharge the GPS logger overnight. In addition, children were asked to record the times and reasons why they took off the devices in a diary. After measurement, devices were collected by the research-staff during school hours, while the child and one of their parents received a verbal and written invitation for an electronic questionnaire.

### Measurement

#### Socio-demographic measures

At baseline, directly after PA measurement, children and parents filled in an electronic questionnaire focusing on aspects such as birth date, address, perceived physical environment, child’s PA, transportation habits and homework (response rate 77 and 75% for the children and parents, respectively). We assessed whether children from divorced parents were residing at two locations, and whether children potentially moved home between baseline and follow-up measurements. Schools provided detailed class timetables for the data-collection period.

#### Accelerometer and GPS loggers

In this study, accelerometers (GT3X, ActiGraph, Pensacola, Florida) were set to record data at 10 s epochs. Actilife version 6.11.9 was used for initialization and downloading. The GPS logger used in this study (BT-Q1000XT, Qstarz International Co, Taipei, Taiwan) showed relatively good static spatial accuracy compared to other units [44], and acceptable dynamic accuracy [45]. The manufacturer’s software QTravel version 1.46 was used for initialization and downloading of GPS loggers. The loggers were set to record data at 10 s epochs while recording parameters such as date, time, longitude, latitude, and speed. GPS devices stopped logging data when storage capacity was full [22].

### Data analysis

#### Data management

Accelerometer and GPS data were processed using the Personal Activity and Location Measurement System (PALMS), which allows users control over most parameter settings in a web-based application [41, 46]. In order to handle the data-load, data were processed in PALMS separately for each school and time-point (i.e. baseline and follow-up). PALMS categorized intensity of accelerometer activity into sedentary time (ST), light PA (LPA), moderate PA (MPA), and vigorous PA (VPA) according to Evenson’s cut-points [47], which are based on free-living activities of 5–15 year-old children [48]. We defined non-wear time as ≥20 consecutive minutes of zero counts [49]. Datasets were cleaned based on extreme speed (i.e. threshold ≥130 kmph) and changes in elevation (i.e. threshold ≥1000 m). Algorithms as described in Carlson et al. (2015) were used for trip and trip mode classification (e.g., pedestrian, bicycle) [40]. As the sample in the study of Carlson et al. (2015) consisted of commuting cyclists that were expected to accumulate higher cycling speeds, the present study deviated from these thresholds by using the 10–25 kmph bicycling speed-threshold. Invalid GPS points were imputed from the last known valid point, for up to 10 min. Finally, PALMS matched accelerometry and GPS data based on start- and end-times of the GPS logger. Subsequently, we combined school-specific datasets into multiple PostgreSQL databases (http://www.postgresql.com), where we performed additional queries to identify before school (i.e. 6: 00 AM – start school time), during school- (i.e. based on individual school’s schedules), and after school time-segments (i.e. end school time – 11:59 PM). Additionally, we also included data on weekend days. This resulted in eight time-segmented datasets (i.e. before school, during school, after school and weekend days at both primary and secondary school), where days were the unit of analyses.

#### Spatial analyses

Time-segmented datasets were integrated into ArcGIS version 10.4.1 (ESRI, Redlands, California), where we overlaid GIS-data from the municipality of ‘s-Hertogenbosch. We used a stepwise selection procedure to identify data-points occurring in various contexts, based on the GPS-derived contexts of Klinker et al. (2014) [22] and the Sensewear-derived contexts of De Baere et al. (2015) [16]. First, we identified each child’s home and school parcel, using ArcGIS geocode-functionality and the municipality’s address-database (see Additional file 1). We then identified the context ‘home’ and ‘school’ by selecting data-points (i.e., records) that were within 10 m of each child’s home-parcel. Secondly, from the remaining records > 10 m from a child’s home and school parcels, we identified the other contexts by selecting data-points within 10 m from sports grounds, commercial/shopping centers or malls, and afterschool care parcels. Third, from the remaining records > 10 m from these more specific parcels, we applied the above-described PALMS speed-thresholds in order to identify transport-related PA. Fourth, records that were not selected in the above stepwise procedure, were defined as records at other locations (e.g. at friend’s homes or at parks).

#### Data reduction

**Fig. 1:**
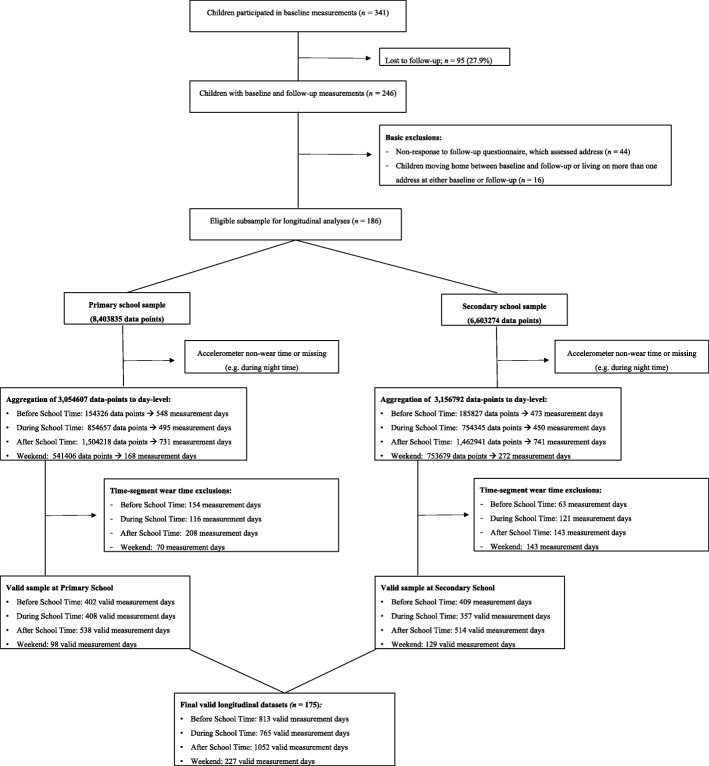
Data flowchart

First, we selected children with longitudinal follow-up data in the transition from primary to secondary school (Fig. 1). Subsequently, data from children that moved home between baseline and follow-up measurements, and with more than one home environment (often in the case of co-parenting) was omitted. Data-points with extremely high accelerometer counts (i.e. > 9498 counts per minute) were also omitted. Subsequently, raw data-points were aggregated into daily totals, segregated by specific time-segments of the day (Fig. 1). As preliminary inspection of the data showed that children only spent little time at afterschool childcare and at commercial/shopping centers, we re-defined these contexts as time spent in other locations. In accordance with previous studies investigating time-segmented PA [50–52], we ensured reliability of the time-segments during school, afterschool and weekend by only selecting records from days with ≥ 50% of the potential wear time. For example, for children from schools that end at 4:00 PM, potential afterschool wear time is 8 hours (as data logging starts ends at 11:59 PM). This results in wear time criteria of at least 240 min of wear time in this specific time segment. Time before school data was included when at least 30 min of combined GPS and accelerometer data was available in that time segment, as this was considered the minimum amount of time children need to prepare for, and travel to school [53]. Finally, we combined baseline and follow-up data in four longitudinal datasets (i.e., before school, during school, after school and at weekend days; Fig. 1).

#### Statistical analyses

After describing changes in total PA in the transition between primary and secondary school, we conducted analyses on the average number of minutes for the time that children contributed to context-specific light PA (LPA) and moderate-to-vigorous PA (MVPA), both in primary school and secondary school. Days with no accelerometer data on specific contexts (e.g., at school during weekends) were assigned zero minutes of PA to that context. In subsequent multivariate models, days were used as the unit of analyses and the main explanatory variable was the index-variable that represented either baseline- or follow-up measurement. All multivariate analyses were performed using longitudinal multilevel linear mixed models, acknowledging the two-level structure of primary-secondary school transition within multiple repeated days and repeated days within children. We modeled this within-subject change between primary and secondary school by adjusting for the interaction between the index-variable defining baseline- or follow up measurements, a variable counting the number of repeated days of measurement within a child and the unique identifying variable for each child. No further covariance between days within children was assumed. Furthermore, a random slope variable was used to account for clustering of children within schools, using a variance component covariance structure. Outcome variables were minutes of LPA and MVPA (specified for each time-segment and context). Model fit and normality of residuals of these multivariate models were inspected to verify its fitting capabilities. In contexts with a relative overrepresentation of zero counts due to non-wear time, non-parametric negative binomial models were applied to estimate change in minutes of LPA and MVPA between primary- and secondary school. However, some contexts still consisted of too few data-points (e.g. during weekend days at the school’s parcel) and were therefore not presented.

Children sometimes reported that they removed devices at random activities throughout the day because they thought the devices were uncomfortable, they were afraid of damaging the devices or when they stayed at home late in the evening. As these activities were not specifically related to PA or inactivity, we hypothesized that non-wear time may not be significantly different from wear time. In addition, we were interested in the total duration of PA patterns performed at specific contexts and whether these patterns would change during the transition. Therefore, we adjusted our multivariate analyses for total daily wear time instead of context-specific wear time. Based on registries from local weather stations, we extracted hourly meteorological data for each day of PA measurement. We computed daily averages of meteorological data, and we adjusted for these meteorological circumstances in subsequent multivariate analyses. Statistical analyses were performed using SPSS 21.0 for Windows (IBM SPSS Inc., Armonk, NY), and *p* < 0.05 indicated statistical significance.

## Results

### Participant characteristics and development of total PA

From the 341 participating children in primary school, 246 children (72%) also participated 1 year later in secondary school. From these children, 186 (75,6%) filled in the questionnaire in secondary school and did not move home (Fig. 1). In total, valid data were provided by 89 boys and 86 girls who attended 20 primary schools and 10 secondary schools. Children were averagely aged 12.1 (SD = 0.4) years old at baseline. Parents from participating children had relatively high socio-economic status; 72% with secondary vocational or higher educational diploma, and 67.9% with a paid job for at least 3 days per week. On average, children lived 620 (SD = 571) meters from their primary school and 3127 (SD = 1976) meters from their secondary school. Based on the standardized social economic status score of 0.06 (SD = 1.3) neighborhood SES of our participants were comparable with the national average [37].

While attending primary schools, children performed more time in MVPA during weekend days compared to weekdays (55.1 versus 43.9 min/day, respectively). Total MVPA declined in secondary school, both during weekend days (from 55.1 to 34.3 min/day), and during weekdays (from 43.9 to 34.0 min/day). Comparable declines were found in LPA. On weekdays, boys exhibited significantly more MVPA at both primary-and secondary school compared to girls, while girls performed more LPA at primary school only. No significant gender differences were found on weekend days (Table 1). When looking at more specific PA domains, declines from primary to secondary were predominantly found in the afterschool period. Namely, children exhibited 27.7 min of MVPA (6.8% from afterschool weartime) in primary-, while 18.4 min (4.4%) were accumulated in secondary school. Smaller declines were found for MVPA during school time (15.5 versus 13.6 min/day). In contrast to this general decline of PA in secondary school, we found that especially LPA increased before school time (30.4 versus 39.0 min/day). Boys exhibited significantly more MVPA during- and afterschool time than girls, both at primary and secondary school. Also, boys showed significantly more LPA before- and during school time, in primary school only. This was partly different for girls, who exhibited significantly more LPA afterschool time at primary and secondary school, and more LPA before school time, in secondary school only (Table 1).

**Table 1:** Total PA in Primary and Secondary School

		Weekend days		Weekdays ^a^		Weekdays Before School Time ^b^		Weekdays During School Time ^b^		Weekdays After School Time ^b^	
		Primary School	Secondary School	Primary School	Secondary School	Primary School	Secondary School	Primary School	Secondary School	Primary School	Secondary School
Total PA (daily mean minutes; SD)	*n* days with wear time	98	129	198	162	403	410	427	367	542	515
	wear time; mean minutes	706.6 (94.4)	692.9 (90.3)	798.7 (75.3)	829.1 (73.4)	57.9 (18.0)	73.4 (27.7)	320.8 (73.7)	328.7 (65.8)	402.5 (78.7)	414.9 (82.8)
	LPA; mean minutes and percentage from wear time	317.6 (87.1); 44.7%	272.2 (94.7); 38.9%	334.7 (82.9); 41.8% ^d^	306.8 (74.0); 37.0%	30.4 (10.3); 52.5% ^c^	39.0 (14.9); 53.1% ^d^	114.6 (46.3); 35.7% ^c^	107.2 (46.3); 32.6% ^d^	175.7 (56.2); 43.7%	155.7 (55.0); 37.5%%
	MVPA; mean minutes and percentage from wear time	55.1 (48.6); 7.6%	34.3 (34.8); 5.0%	43.9 (32.3); 5.5% ^c^	34.0 (32.1); 4.1% ^c^	2.9 (2.6); 5.0%	3.5 (4.7); 4.8%	15.5 (13.7); 4.8% ^c^	13.6 (19.4); 4.1% ^c^	27.7 (24.2); 6.8% ^c^	18.4 (23.1); 4.4% ^c^

^a^based on smaller subsample of days with valid data in both before- during- and after school time. ^b^: based on individual samples that included days with data on any behavioural domain or any context, irrespective of other domains or contexts. Gender differences were analyzed using multi-level linear mixed models adjusted for gender, age, number of days with valid PA measurement, and clustering of days within children and children within schools. ^c^: significantly higher for boys compared to girls. ^d^: significantly higher for girls compared to boys

### Context-specific PA patterns in primary and secondary school

During school time, children spent more time outside school grounds in secondary school, while at primary school, children spent more time on the school grounds. On school grounds, the relative intensity of PA was stable, while outside school grounds percentages of LPA and MVPA declined. In the afterschool period, time spent at home increased with an average of about 50 min in secondary school, while LPA and MVPA performed at home was relatively stable. During afterschool periods, sports grounds were visited less frequently, leading to an average decline of approximately 8.5 min of wear time per day at sport grounds, which in turn resulted in declines of both LPA and MVPA (12.3 versus 9.6 min/day and 6.5 versus 4.6 min/day, respectively). In addition, time spent in LPA at other locations decreased with approximately 25 min in the afterschool period, as did MVPA (9.5 versus 5.1 min/day). During weekend days, relatively small declines in both LPA and MVPA were found across various locations (Table 2).

**Table 2:** Context-specific Physical Activity patterns in Primary and Secondary School

		Home		School grounds		Sports grounds		Other locations	
		Primary school	Secondary School	Primary school	Secondary School	Primary school	Secondary School	Primary school	Secondary School
Before School Time (daily mean minutes; SD)	*n* days with valid wear time	402	409	402	409	^a^	^a^	^a^	^a^
	wear time; mean minutes	35.5 (18,8)	36.0 (22.4)	8.6 (6.8)	14.8 (17.0)	^a^	^a^	^a^	^a^
	LPA; mean minutes and percentage from context-specific wear time	17.7 (9.7); 49.9%	17.5 (10.0); 48.6%	5.3 (4.0); 61.6%	7.2 (7.2); 48.6%	^a^	^a^	^a^	^a^
	MVPA; mean minutes and percentage from context-specific wear time	0.5 (0.8); 1.4%	0.4 (1.2); 1.1%	0.7 (1.5); 8.1%	1.1 (3.0); 7.4%	^a^	^a^	^a^	^a^
During School Time (daily mean minutes; SD)	*n* days with valid wear time	^a^	^a^	427	367	^a^	^a^	427	367
	wear time; mean minutes	^a^	^a^	238.3 (107.2)	205.0 (118.2)	^a^	^a^	35.7 (49.4)	98.0 (130.2)
	LPA; mean minutes and percentage from context-specific wear time	^a^	^a^	79.2 (47.1); 33.2%	64.1 (44.4); 31.2%	^a^	^a^	13.9 (18.7); 38.9%	28.0 (38.1); 28.6%
	MVPA; mean minutes and percentage from context-specific wear time	^a^	^a^	7.1 (10.4); 3.0%	5.7 (8.2); 2.7%	^a^	^a^	2.3 (4.5); 6.4%	3.6 (12.8); 3.7%
After School Time (daily mean minutes; SD)	*n* days with valid wear time	542	515	542	515	542	515	542	515
	wear time; mean minutes	200.0 (105.0)	248.7 (113.7)	18.3 (38.2)	20.9 (47.1)	24.2 (44.0)	15.6 (39.1)	140.4 (106.6)	113.5 (114.7)
	LPA; mean minutes and percentage from context-specific wear time	76.6 (43.6); 38.3%	82.5 (43.4); 33.2%	10.8 (20.0); 59.0%	10.2 (21.1); 48.8%	12.3 (23.5); 50.8%	7.6 (19.7); 48.7%	58.0 (49.9); 41.3%	38.7 (42.7); 34.1%
	MVPA; mean minutes and percentage from context-specific wear time	3.3 (4.8); 1.7%	2.6 (4.1); 1.0%	2.4 (7.3); 13.1%	1.4 (3.8); 6.7%	6.5 (15.1); 26.9%	4.6 (14.5); 29.5%	9.5 (14.2); 6.8%	5.1 (10.8); 4.5%
Weekend days (daily mean minutes; SD)	*n* days with valid wear time	98	129	^a^	^a^	98	129	98	129
	wear time; mean minutes	319.6 (176.0)	342.8 (187.0)	^a^	^a^	41.3 (86.8)	26.7 (71.2)	315.2 (192.1)	317.2 (253.2)
	LPA; mean minutes and percentage from context-specific wear time	139.1 (81.3); 43.5%	125.1 (70.6); 36.5%	^a^	^a^	20.2 (44.5); 48.9%	13.8 (36.4); 51.7%	124.9 (78.5); 39.6%	111.4 (87.7); 35.1%
	MVPA; mean minutes and percentage from context-specific wear time	9.5 (11.7); 3.0%	6.0 (10.1); 1.7%	^a^	^a^	9.9 (23.0); 24.0%	5.6 (19.6); 21.0%	23.8 (24.9); 7.6%	14.5 (18.9); 4.6%

^a^: insufficient variation to calculate daily mean minutes. Gender differences were analyzed using multi-level linear mixed models adjusted for gender, age, number of days with valid PA measurement, and clustering of days within children and children within schools. ^b^: significantly higher for boys compared to girls. ^b^: significantly higher for girls compared to boys

### Transport-related (in) activity patterns in primary and secondary school

We identified records as transport-related (in) activity when 1) records occurred > 10 m from home, school, sports grounds, commercial/shopping centers or afterschool care parcels, and 2) records met the GPS-based trip detection algorithms as either active transport (i.e., walking or cycling) or passive transport. Before school time, active transport increased in secondary school, which led to an increase in LPA (3.8 versus 11.5 min/day). In contrast, transport-related PA remained relatively stable during- and afterschool time. During weekend days however, we found a decline in transport-related LPA (19.4 versus 10.1 min/day) and MVPA (11.2 versus 7.6 min/day). We also found small declines in the use of passive transport in the transition period. Gender differences were found in the afterschool period, where girls showed significantly more LPA in active transport, whereas boys showed significantly more MVPA in active transport (Table 3).

**Table 3:** Transport-related (in) activity patterns in Primary and Secondary School

		Active transport		Passive transport	
		Primary school	Secondary School	Primary school	Secondary School
Before School Time (daily mean minutes; SD)	*n* days with valid wear time	403	410	403	410
	wear time in transport; mean minutes	3.8 (3.8)	11.5 (8.5)	0.9 (3.6)	0.8 (3.0)
	LPA; mean minutes and percentage from transport-related PA	2.8 (3.3); 73.7%	8.9 (7.6); 77.4%	0.4 (1.4); 44.4%	0.3 (1.4); 37.5%
	MVPA; mean minutes and percentage from transport-related PA	0.7 (1.5); 18.4%	0.7 (2.2); 6.1% ^c^	a	a
During School Time (daily mean minutes; SD)	*n* days with valid wear time	427	367	a	a
	wear time in transport; mean minutes	8.0 (11.4)	7.2 (11.8)	a	a
	LPA; mean minutes and percentage from transport-related PA	4.5 (6.7); 56.3%	5.2 (8.6); 72.2%	a	a
	MVPA; mean minutes and percentage from transport-related PA	1.7 (3.4); 21.3%	1.1 (4.6); 15.3%	a	a
After School Time (daily mean minutes; SD)	*n* days with valid wear time	542	515	542	515
	wear time in transport; mean minutes	21.1 (19.8)	20.8 (21.8)	10.9 (19.6)	7.9 (19.5)
	LPA; mean minutes and percentage from transport-related PA	13.4 (13.5); 63.5%	14.3 (14.4); 68.8% ^c^	4.7 (8.9); 43.1%	3.0 (6.8); 38.0% ^c^
	MVPA; mean minutes and percentage from transport-related PA	5.1 (8.3); 24.2%	4.4 (11.9); 21.2% ^b^	a	a
Weekend days (daily mean minutes; SD)	*n* days with valid wear time	89	129	89	129
	wear time in transport; mean minutes	35.1 (34.9)	18.7 (21.8)	36.1 (47.1)	35.8 (49.8)
	LPA; mean minutes and percentage from transport-related PA	19.4 (26.1); 55.3%	10.1 (13.3); 54.0%	14.2 (19.3); 39.3%	12.2 (16.4); 34.1%
	MVPA; mean minutes and percentage from transport-related PA	11.2 (16.0); 32.1%	7.6 (13.6); 40.6%	a	a

^a^: insufficient variation to calculate daily mean minutes. Gender differences were analyzed using multi-level linear mixed models adjusted for gender, age, number of days with valid PA measurement, and clustering of days within children and children within schools. ^b^: significantly higher for boys compared to girls. ^c^: significantly higher for girls compared to boys

### Changes in context-specific PA patterns and transport-related (in) activity from primary to secondary school

In Table 4, we explained changes of children’s context-specific PA patterns between primary and secondary school using multivariate models. Before school, children accumulated 6.34 more daily LPA minutes in active transport. During school, total LPA declined with approximately 11 min/day. More specifically, we found that LPA and MVPA at other locations increased in secondary school (14.39 min/day and 2.38 min/day, respectively). In contrast, LPA at school grounds decreased during school (− 18.96 min/day). In afterschool periods, total LPA and MVPA declined (− 25.04 min/day and − 7.98 min/day, respectively). More specifically, LPA and MVPA decreased significantly at other locations (− 21.20 min/day and − 5.20 min/day, respectively). Furthermore, we found small decreases in LPA at sports grounds (− 3.54 min/day) and MVPA at school grounds (− 1.05 min/day). During weekend days, the above described declines of total LPA and MVPA were no longer statistically significant in the adjusted model. Furthermore, LPA in active transport decreased with 15.88 min during an average weekend day, while changes in transport related MVPA were not significant. Children’s passive transport slightly decreased before- and after school (− 0.43 min/day and − 1.65 min/day) (Table 4).

**Table 4:** Changes in context-specific Physical Activity and transport-related (in) activity patterns in transition between Primary to Secondary School

		All locations and domains crude ^a^	All locations and domains adjusted ^b^	Context-specific PA ^b^				Transport-related PA ^b^	
				Home	School grounds	Sports grounds	Other locations	Active transport	Passive transport
Before School Time (daily mean minutes; SD) *n* days with valid wear time = 813	LPA; mean minutes	2.71 (1.09 to 4.33)	2.36 (0.46 to 4.25)	**−3.43 (−5.31 to − 1.54)**	0.55 (−0.57 to 1.67)	c	c	**6.34 (5.27 to 7.42)**	**−0.43 (−0.72 to − 0.14)**^d^
	MVPA; mean minutes	0.22 (−0.48 to 0.92)	0.09 (− 0.74 to 0.92)	− 0.18 (− 0.39 to 0.02)	0.34 (− 0.10 to 0.78)	c	c	− 0.02 (− 0.43 to 0.39)	c
During School Time (daily mean minutes; SD) *n* days with valid wear time = 794	LPA; mean minutes	**−11.92 (−18.94 to − 4.90)**	**−11.02 (−19.45 to − 2.61)**	c	**−18.96 (−29.02 to − 9.51)**	c	**14.39 (8.15 to 20.63)**	1.17 (− 0.57 to 2.92) ^d^	c
	MVPA; mean minutes	−1.41 (−4.42 to 1.61)	2.78 (− 0.65 to 6.22)	c	−0.29 (− 2.34 to 1.76)	c	**2.38 (0.76 to 4.00)**	−0.45 (− 1.33 to 0.44) ^d^	c
After School Time (daily mean minutes; SD) *n* days with valid wear time = 1057	LPA; mean minutes	**− 26.80 (− 33.95 to − 19.64)**	**−25.04 (− 32.81 to − 17.27)**	1.67 (−6.09 to 9.43)	−0.42 (− 3.86 to 3.02) ^d^	**− 3.54 (−7.08 to − 0.01)**^d^	**− 21.20 (− 29.02 to − 13.38)**	1.81 (−0.58 to 4.21)	**− 1.65 (− 2.93 to − 0.38)**^d^
	MVPA; mean minutes	**−9.54 (− 13.18 to − 5.90)**	**− 7.98 (− 12.05 to − 3.91)**	− 0.51 (− 1.31 to 0.29)	**−1.05 (− 1.92 to − 0.19)**^d^	−0.57 (− 3.00 to 1.86) ^d^	**− 5.20 (− 7.33 to − 3.07)**	− 0.59 (− 2.68 to 1.51)	c
Weekend days (daily mean minutes; SD) *n* days with valid wear time = 227	LPA; mean minutes	**− 38.36 (− 58.36 to − 18.37)**	−30.27 (− 66.71 to 6.17)	10.27 (− 28.47 to 49.00)	c	−17.41 (− 38.14 to 3.32) ^d^	3.19 (−37.36 to 43.74)	**−15.88 (− 25.21 to − 6.54)**	−2.43 (− 12.01 to 7.16) ^d^
	MVPA; mean minutes	**−16.79 (− 27.54 to − 6.04)**	0.31 (− 19.95 to 20.57)	0.05 (− 5.75 to 5.85)	c	−2.29 (− 13.21 to 8.63) ^d^	−1.51 (− 12.69 to 9.67)	0.65 (− 7.16 to 8.47)	c

Dependent variables are minutes of LPA and MVPA in each context or domain. ^a^: multi-level linear mixed models only adjusted for total daily wear time and clustering of days within children and children within schools. ^b^: multi-level linear mixed models also adjusted for gender, age, number of days with valid PA measurement, and meteorological variables. ^c^: insufficient variation in data points to calculate multilevel model (see Tables 2 and 3). ^d^: multi-level non-parametric negative binomial models also adjusted for gender, age, number of days with valid PA measurement, and meteorological variables. Bold numbers represent statistical significance at *p* < 0.05

## Discussion

To our knowledge, this was the first study using combined accelerometer, GPS and GIS data to investigate longitudinal associations of PA patterns in the transition phase from primary to secondary school, in order to add in-depth insights of how, where and when changes occur. We found a substantial decline of PA in transition from primary to secondary school. The decline however did not occur evenly across daily time periods and locations. Whereas total daily PA declined, transport-related PA during weekdays increased. Children showed notable declines in PA performed at sports grounds and at other locations.

Several results were in line with findings of previous studies, which were based on self-reported or device-assessed data. First, in line with studies reporting increases in overall self-reported transport-related PA in transition to secondary school [10, 13, 15, 16], we also found an increase in time spent in active transport, especially before school. This increase in transport-related PA may be a result of parents providing these children with more independent mobility as they get older. However, as in our sample we found that transport-related PA significantly declined during weekends in secondary school, increased transport-related PA in our sample was probably related to the increased distance between home and school. Children with increased transport-related PA during weekdays may have compensated this with declines of afterschool LPA at other locations or with declining participation in organized sports. As investigating compensation mechanisms was not the primary purpose of this paper, future research using detailed within-person designs and larger samples are needed to unravel compensation mechanisms in context-specific PA patterns. The present study contributes by providing in-depth insights in changes of daily PA patterns in transition between primary-and secondary school, in terms of location, time and domain-specific behaviour [13, 17, 54, 55]. Furthermore, this study shows that when investigating determinants of PA patterns in school-aged children, weekday and weekend day patterns differ, for example a by varying contribution of transport-related PA to their PA pattern. This is in line with theories of space-time geography and activity-space approach, which contemplate that PA patterns are partly shaped by time-constraints, social influences and by exposure to environments attributes in their own activity-space [24, 36]. As the activity-space and time-constraints of children during weekends may be fundamentally different from weekdays, segregation of these days is vital in understanding determinants of children’s PA patterns. In addition, future studies are encouraged to include more meaningful behavioural domains using geographical, cultural or temporal contexts, in order to understand changes in PA patterns and to foster international comparisons of PA-promoting initiatives.

Second, we found that total LPA during school time declined in secondary school, which supports conclusions of a 3-year follow-up study of Harding et al. (2015) [18] who reported parallel decreases of LPA during school, after school, and in weekends, but contrasts results of a 4-year follow-up study of Brooke et al. (2014) [20]. Also, the number of minutes spent in MVPA during school hours was comparable with previous studies in this age group [16, 56]. Our study showed that during school hours, more time was spent outside school grounds. This resulted in increased LPA and MVPA outside school grounds but on the other hand decreased LPA at school grounds. This may be related to children performing educational activities outside the school’s geographical parcel, such as surrounding neighborhoods or parks. Although the relative contribution to daily total MVPA during weekdays was slightly higher, we found a decline in the average minutes of MVPA that children spent at schoolgrounds. As emerging evidence links daily PA to academic performance [57–59] and when taking into account the general decline of PA across contexts and locations, schools are encouraged to utilize their potential for increasing PA during- and after school hours at school grounds, for example by providing physical education or afterschool PA opportunities. When attending primary school, but especially after the transition to secondary school, we found that sports grounds are also important in the accumulation of MVPA. This stresses the need to prevent further drop out in sports participation in secondary school children to prevent further decreases in MVPA during adolescence.

Third, in line with our finding that total LPA and MVPA declined in the transition-period, we found that afterschool LPA and MVPA also declined in secondary school, which supports conclusions from three studies [14, 18, 20]. These findings are also in line with self-reported data of three studies that suggested increased time spent on homework, leisure time TV and computer use in transition to secondary school [15–17]. Our study adds that changes in after school PA were predominantly due to a decrease of PA performed at home and other locations (e.g. at friend’s homes or at parks).

### Strengths and weaknesses

A study strength is the utilization of GPS devices in order to investigate associations between the device-assessed context-specific PA patterns. Moreover, the longitudinal design, as well as the in-depth analyses of a naturally occurring, important transition in the development of children’s PA patterns may be considered a strength of the study. However, this study also has some weaknesses. First, in order to reduce complexity of associations, analyses were conducted on a subsample (i.e. non-movers and living at a single address). Due to period-specific wear time validation criteria and by the specificity of our context-specific analyses, the amount of measurement-days in our analyses was further reduced. This limited our statistical power, especially in multilevel models. Attrition analyses revealed that total PA of our final sample was generally comparable with larger sample of children providing device-assessed data on any day, both in terms of cross-sectional and longitudinal comparisons. Only the average minutes of LPA and MVPA performed during weekend days and LPA performed after school was slightly higher in our final subsample compared to the larger sample (Additional file 3). Another important issue in analyzing context-specific PA data is that by theory, a child’s daily individual PA pattern consists of multiple interacting contexts. As a consequence, increases of PA spent in one context by definition results in fewer time/opportunities to perform PA in another context [60]. In contrast to this more individually based compositional approach, the present study applied a more context-centered approach to provide insights of how children’s general PA patterns performed at various contexts change during transition from primary to secondary school.

Another limitation of this study was the specificity of our time-segment during school. This may be improved by also defining recess time and/or physical education lessons [20, 61]. As we found that children spent considerable time outside school grounds during school time, more specific information regarding activities during recess, or trips to externally located physical education facilities may have improved our understanding of these behaviours. Finally, we found that active transport plays an important role in reducing the negative impact of PA in secondary school. In contrast to findings of other studies [62, 63], increases in active transport predominantly resulted in higher LPA (and not MVPA). This may be a result of relatively higher contribution of cycling trips to total transport-related PA instead of walking trips (due to increased distance between home and school) and the tendency of our hip-worn ActiGraph GT3X+ accelerometers to underestimate the workload of especially cycling trips [64]. Furthermore, future studies may investigate transport-related PA patterns even more specifically by identifying start- and endpoints of a trip. For example, such studies could specifically investigate determinants of children’s daily active- versus passive transportation modes between home and school [62].

Generalizability of this study may be limited to samples with comparable cultural and infrastructural circumstances. For example, in the Netherlands, there are no organized passive transport programs in place (e.g. school busses) and the environment is generally supportive for active transport (e.g. absence of hills, high availability and quality of cycling paths, bike sheds at schools). This may increase the likelihood of active transport in the home-school commute, regardless of the distance between home and school. In addition, parental motives regarding independent mobility and accompanied feeling of neighbourhood safety may be different from other international samples [65]. Also, time-constrains of children due to competing activities such as organized sports participation or homework may also be considered in comparing results with other studies [36].

## Conclusion

This is the first study that demonstrated device-assessed longitudinal changes of context- and domain-specific PA patterns in transition between primary and secondary school. We found that overall PA significantly declined from primary to secondary school. Furthermore, we showed that although transport-related PA increased before and during school, especially afterschool PA spent sports grounds, afterschool PA spent at other locations, and transport-related PA during weekends decreased in transition from primary to secondary school. Given the importance of this transition-period for the development of long-term PA patterns, results from this study warrant the development of evidence-based PA programs in this transition period, while acknowledging the integrative role of schools, parents, and afterschool sports providers. More specifically, we emphasize the need to increase children’s PA levels in primary schools, promote afterschool PA at secondary schools, and to prevent the drop-out in sports participation at secondary schools.

## Supplementary information

**Additional file 1.** Geographical distribution of participating primary and secondary schools in the municipality of ‘s-Hertogenbosch, the Netherlands.

**Additional file 2.** STROBE checklist.

**Additional file 3.** Attirition analyses.

## Data Availability

The datasets used and/or analyzed during the current study are available from the corresponding author on reasonable request.

## Abbreviations

PA: Physical activity
ST: Sedentary time
LPA: Light physical activity
MPA: Moderate physical activity
VPA: Vigorous physical activity
MVPA: Moderate-to-vigorous physical activity
GPS: Global positioning system
GIS: Geographical information system
PALMS: Personal Activity and Location Measurement System
PHASE: Physical activity in public space environments
