# Supplementary figures and images for: Investigating longitudinal context-specific physical activity patterns in transition from primary to secondary school using accelerometers, GPS, and GIS
Source: Int J Behav Nutr Phys Act. 2020 May 18;17:66. doi: 10.1186/s12966-020-00962-3 (PMC7236458; doi:10.1186/s12966-020-00962-3)

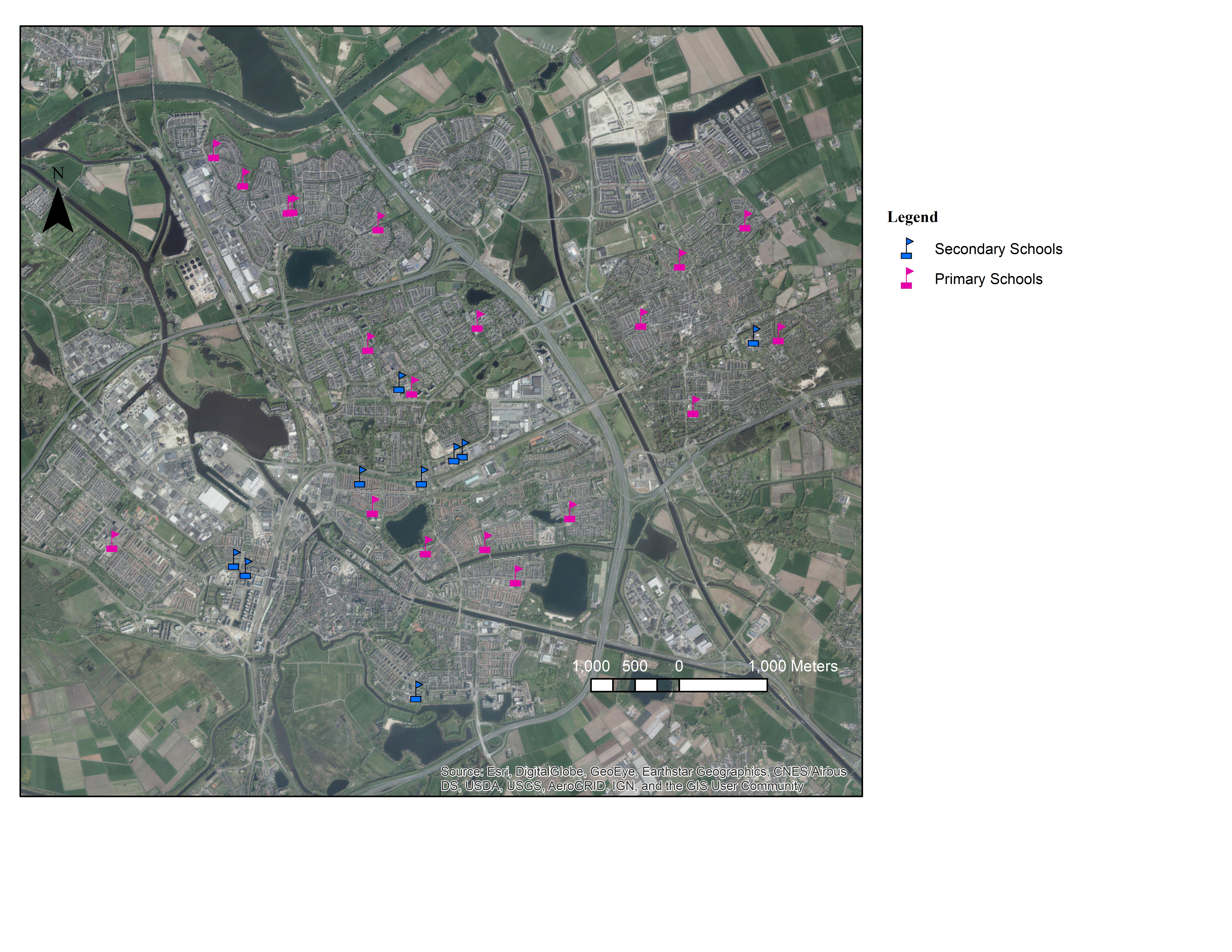

Supplement: Supplementary file 1 — Additional file 1. Geographical distribution of participating primary and secondary schools in the municipality of ‘s-Hertogenbosch, the Netherlands. [file 12966_2020_962_MOESM1_ESM.jpg]
