# Supplementary material for: Investigating longitudinal context-specific physical activity patterns in transition from primary to secondary school using accelerometers, GPS, and GIS
Source: Int J Behav Nutr Phys Act. 2020 May 18;17:66. doi: 10.1186/s12966-020-00962-3 (PMC7236458; doi:10.1186/s12966-020-00962-3)
Supplement: Supplementary file 3 — Additional file 3. Attirition analyses. [file 12966_2020_962_MOESM3_ESM.docx]

Additional file 2: Attrition analyses comparing the final sample presented in the manuscript with the larger sample of children providing device-assessed data at any day.

|  |  | Sample in paper | | | Total sample | | |
| --- | --- | --- | --- | --- | --- | --- | --- |
|  |  | Primary school | Secondary school | Difference Primary-Secondary (%) | Primary school | Secondary school | Difference Primary-Secondary (%) |
| Weekdays Before School Time | LPA | 28,3 | 38,0 | 34,3% | 25,3 | 35,6 | 40,7% |
|  | MVPA | 2,1 | 2,4 | 14,3% | 2,9 | 3,3 | 13,8% |
| Weekdays During School Time | LPA | 113,4 | 105,0 | -7,4% | 104,1 | 94,1 | -9,6% |
|  | MVPA | 13,2 | 10,9 | -17,4% | 14,4 | 13,1 | -9,0% |
| Weekdays After School Time | LPA | 174,7 | 155,7 | -10,9% | 150,6 | 126,1 | -16,3% |
|  | MVPA | 26,8 | 18,4 | -31,3% | 24,7 | 16,8 | -32,0% |
| Weekend days | LPA | 317,6 | 272,2 | -14,3% | 240,3 | 180,0 | -25,1% |
|  | MVPA | 55,1 | 34,3 | -37,7% | 49,4 | 27,7 | -43,9% |
